# Capsule Endoscopy for the Risk Stratification and Management of Acute Upper Gastrointestinal Bleeding in Emergency Departments: A Systematic Review on Triage, Risk Stratification, and Management

**DOI:** 10.7759/cureus.71530

**Published:** 2024-10-15

**Authors:** Sulaiman M Alamro, Mazi M Alanazi, Wejdan K Suwayyid

**Affiliations:** 1 Department of Medicine, College of Medicine, Qassim University, Buraidah, SAU; 2 Emergency Medicine, King Saud Medical City, Riyadh, SAU

**Keywords:** capsule endoscopy, emergency departments, gastrointestinal bleeding, risk stratification, triage

## Abstract

Gastrointestinal bleeding is a common occurrence in emergency departments. The standard of care for it is an esophagogastroduodenoscopy within 24 hours to diagnose and potentially treat the bleeding. Several tools and pre-endoscopic risk assessment scores are used to help evaluate and manage upper gastrointestinal bleeding. Additionally, capsule endoscopy provides a non-invasive method to visualize the gastrointestinal tract and identify lesions.

The aim of this review was to explore the role of capsule endoscopy in the triage, stratification, and management of upper gastrointestinal bleeding patients in the emergency department.

Four databases, PubMed, Scopus, Embase, and the Cochrane Library, were searched using keywords related to capsule endoscopy and gastrointestinal bleeding in the emergency department. Studies were included if they assessed the use of capsule endoscopy in emergency settings for managing upper gastrointestinal bleeding. After screening titles, abstracts, and full texts and reviewing bibliographies for relevant articles, data on study design, participant demographics, capsule endoscopy procedure description, and outcomes were collected into a pre-designed spreadsheet. The review focused on the triage, risk stratification, management impact, and safety of capsule endoscopy.

The literature search identified 712 records, with 37 studies screened for full-text review, resulting in nine studies included in the review. These studies involved 634 patients and primarily compared capsule endoscopy with standard care in emergency settings. Capsule endoscopy demonstrated potential advantages, including improved identification of high-risk lesions and reduced hospital admissions. Capsule endoscopy effectively detected bleeding and reduced the need for invasive procedures compared to standard practices. Capsule endoscopy also had lower costs relative to traditional triaging methods.

The use of capsule endoscopy in the risk stratification and management of upper gastrointestinal bleeding in emergency settings is promising. Its high diagnostic accuracy can enhance patient outcomes by enabling timely and precise diagnosis, reducing the need for invasive procedures. Future research should focus on larger randomized trials to validate capsule endoscopy's efficacy and explore its cost-effectiveness.

## Introduction and background

Gastrointestinal bleeding (GIB) is a common occurrence in emergency departments (ED) [1]. Upper gastrointestinal bleeding (UGIB) is defined as blood loss originating proximal to the ligament of Treitz suspensory muscle of the duodenum and often presents with symptoms such as hematemesis, melena, hematochezia, or signs of hypovolemic shock [2,3]. The mortality rate for UGIB ranges from 2% to 15% [4]. For patients with suspected UGIB, the standard of care (SOC) is an esophagogastroduodenoscopy (EGD) within 24 hours to diagnose and potentially treat the bleeding [5].

Several tools and pre-endoscopic risk assessment scores, such as the Glasgow-Blatchford score (GBS) and the Rockall score, are used to help evaluate and manage UGIB [6-8]. However, these scores are not consistently applied in clinical practice and have limited utility in predicting and confirming bleeding in suspected cases [6-8]. Consequently, an endoscopic examination is typically necessary for definitive diagnosis and treatment, if needed [6-8].

Capsule endoscopy (CE), introduced by Iddan et al. in 2000, provides a non-invasive method to visualize the gastrointestinal (GI) tract [9]. CE has become widely accepted for various applications, including identifying small intestine mucosal lesions, assessing gastric motility, and evaluating occult bleeding [10-13]. However, little is known regarding the use of CE in the ED. The aim of this review was to explore the role of CE in the triage, stratification, and management of UGIB patients in the ED. 

## Review

Methods 

This systematic review was performed according to the Preferred Reporting Items for Systematic Reviews and Meta-Analyses (PRISMA) guidelines [14]. A systematic search of the literature was performed on four databases: PubMed, Scopus, Embase, and the Cochrane Library. The search was performed using the following keywords: capsule endoscopy, video capsule endoscopy, emergency department, emergency room, emergency service, gastrointestinal bleeding, acute gastrointestinal bleeding, and non-invasive diagnostics. These keywords were combined using Boolean operators, and Medical Subject Headings (MeSH) terms and other filters were used according to the database. No restriction was applied in terms of study design apart from editorials, reviews, commentaries, case reports and series, conference proceedings, and abstracts. The search was restricted to the English language and human subjects.

As for the inclusion criteria, we only included studies that assessed the utilization of CE in emergency settings for the triaging, risk stratification, and management of UGIB. After the removal of the duplicates from the identified records, the titles and abstracts of the remaining records were screened to identify the potentially eligible studies. After that, the full texts of the potentially eligible studies were retrieved and assessed for eligibility. Additionally, the bibliography list of relevant articles was also screened for eligible records. 

Data was extracted into a pre-designed spreadsheet. The extracted data included the study design, country of the study, aim of the study, number of participants, gender of the participants, blood pressure of the participants, average time to EGD, type of the CE used in the studies, patient preparation and CE procedure, outcomes measured in the included articles, and impact on using CE in the ED according to performance measures used in the included studies. We also extracted information regarding the safety and adverse events of using CE. 

Results 

**Figure 1:**
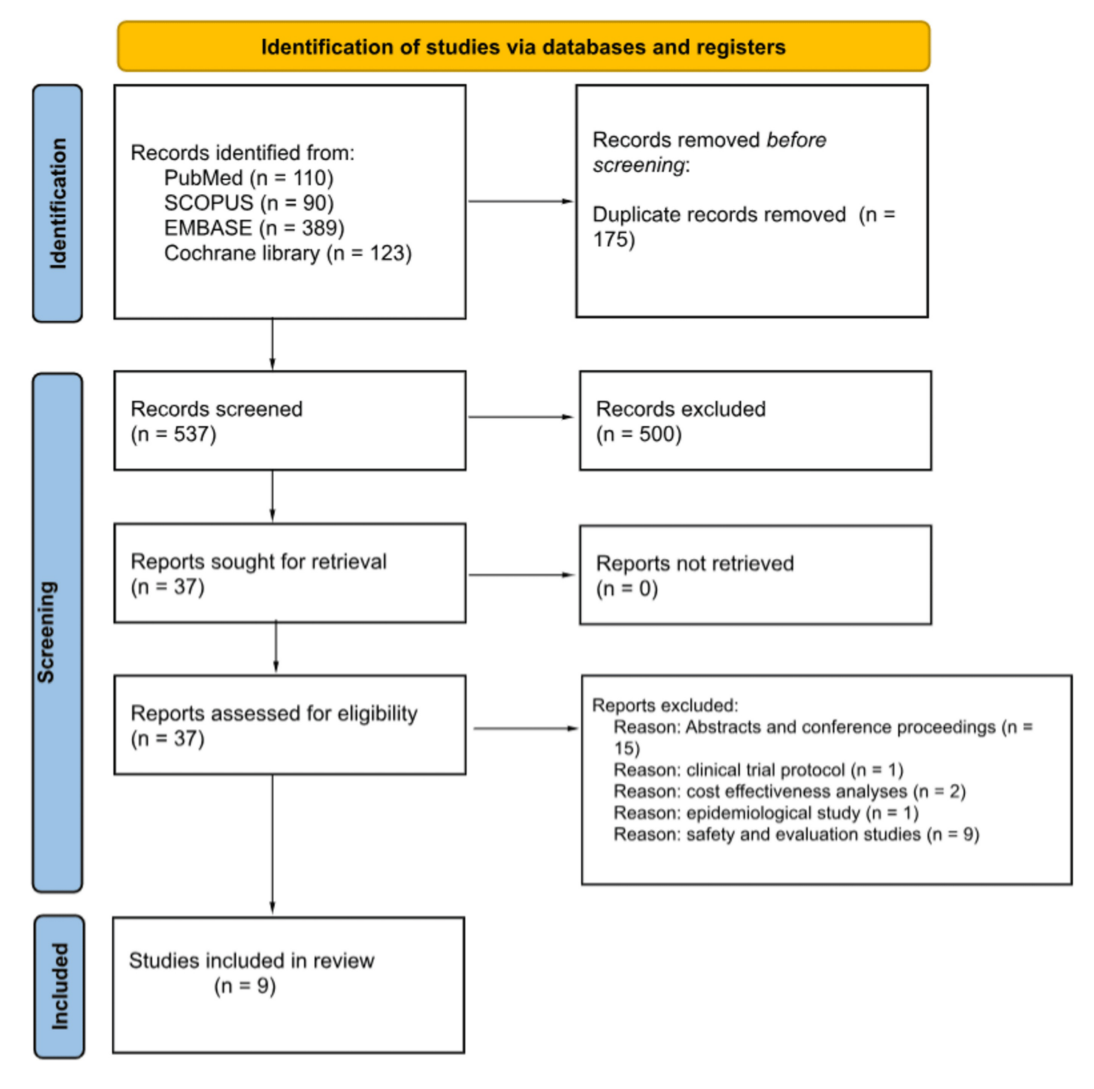
PRISMA flowchart of the study selection process PRISMA: Preferred Reporting Items for Systematic Reviews and Meta-Analyses

The literature search resulted in the identification of 712 records, 175 duplicates were removed, and the titles and abstracts of the rest of the records were screened for inclusion. Of them, 37 entered the full-text screening phase yielding nine studies that were included in this review. Figure 1 demonstrates the study selection process in this review. The majority of the included studies with the exception of two articles had a clinical trial design. Additionally, a total of 634 patients were included in these studies, and the characteristics of the included studies are shown in Table 1. 

**Table 1:** Characteristics of the included studies USA: United States of America; RCT: randomized clinical trial; CT: clinical trial; UGIB: upper gastrointestinal bleeding; CE: capsule endoscopy; EGD: esophagogastroduodenoscopy; M/F: male/female; BP: blood pressure; MAP: mean arterial pressure; SBP: systolic blood pressure; NA: not available; ED: emergency department

Study	Country	Design	Aim	Number of patients	Mean age (years)	Gender (M/F)	Mean/median BP parameters for patients
Rubin et al. 2011 [15]	USA	RCT	To evaluate the effectiveness of CE in accurately identifying high-risk patients by detecting signs of hemorrhage in patients with acute UGIB	24 (12 in the EGD group and 12 in the CE group)	CE: 61.2; EGD: 71.2	15/9	SBP: CE: 121.75; EGD: 125.33
Chandran et al. 2013 [16]	Australia	CT	To determine if an esophageal capsule can identify a low-risk group of patients with UGIB who can safely wait for elective EGD	83	65	65/18	SBP: 124
Gralnek et al. 2013 [17]	China	Cohort study	To assess the feasibility of using CE for patients presenting to the ED with acute overt UGIB	49	58.3	32/17	BP: 137/72
Gutkin et al. 2013 [18]	USA	RCT	To compare the accuracy of the Rockall and Blatchford scores with CE in predicting high-risk endoscopic stigmata	24 (12 in the EGD group and 12 in the CE group)	66	14/10	MAP: CE: 78.47; EGD: 88.22
Sung et al. 2016 [19]	China	RCT	To evaluate if CE can reduce unnecessary hospital admissions in patients with suspected UGIB	71, 34 in each treatment group (CE and standard care)	CE: 55.2; standard care: 54.9	45/23	BP: CE: 132/78; standard care: 133/72
Marya et al. 2019 [20]	USA	RCT	To compare the bleeding localization rates between patients undergoing early CE and those receiving standard care for new-onset non-hemodynamically significant GIB during hospitalization	87 (45 in the standard care group and 42 in the CE group)	65.4	51/36	SBP: CE: 124.4; standard care: 125.3
Hakimian et al. 2021 [21]	USA	Cohort study	To evaluate the effectiveness of CE compared to standard care in triaging patients with GIB	146 (72 in the standard care group and 74 in the CE group)	CE: 64.93; standard care: 61.33	92/54	NA
Meltzer et al. 2021 [22]	USA	RCT	To compare the effectiveness of video CE versus standard care in triaging patients in the ED with UGIB	24 (13 in the standard care group and 11 in the CE group)	CE: 55; standard care: 47	11/13	NA
Akiki et al. 2024 [23]	USA	CT	To evaluate the safety and effectiveness of the PillSense system, a novel swallowed bleeding sensor, in detecting UGIB compared to endoscopy findings	126	62.4	75/51	BP: 130/72

Most of the studies compared CE to the usual SOC in the ED and assessed its risk stratification abilities. The most commonly used type of EC was the PillCam ESO. Intravenous metoclopramide was the dominant choice in the preparation for CE in most of the studies. Table 2 shows the characteristics of CE in the included studies.

**Table 2:** Characteristics of CE in the included studies EGD: esophagogastroduodenoscopy; CE: capsule endoscopy; NA: not available

Study	Type of capsule	Patient preparation	Average time to EGD
Rubin et al. 2011 [15]	PillCam ESO	Intravenous metoclopramide	2.5 hours (high risk classified by CE); 8.4 hours (low risk classified by CE); 8.9 hours in the control group (regular EGD)
Chandran et al. 2013 [16]	PillCam ESO	Intravenous metoclopramide, swallow test (with 100 mL) of water before the ingestion of the capsule	23 hours
Gralnek et al. 2013 [17]	PillCam ESO 2 (modified: battery time increased to at least 90 minutes)	Single dose of intravenous erythromycin (250 mg)	12-24 hours
Gutkin et al. 2013 [18]	PillCam ESO 2	Intravenous metoclopramide	Within 6 hours (high risk classified by CE); within 24 hours (low risk classified by CE and control EGD group)
Sung et al. 2016 [19]	PillCam ESO 2	Patients fasted for at least five hours before the procedure, intravenous metoclopramide	24 hours
Marya et al. 2019 [20]	EC-S10 CE capsule	Intravenous metoclopramide (10 mg) or intravenous erythromycin (125 mg) (only if the capsule remained in the stomach)	Standard of care: 16 hours, CE: 21 hours
Hakimian et al. 2021 [21]	PillCam SB 3	Patients fasted for 6-8 hours before capsule ingestion	NA
Meltzer et al. 2021 [22]	PillCam	Intravenous metoclopramide	NA
Akiki et al. 2024 [23]	PillSense system	Patients were required to fast for a minimum of two hours for clear liquids and four hours for other than a clear liquid diet before ingestion	4 hours after capsule ingestion

Rubin et al.' study aimed to evaluate the effectiveness of CE in accurately identifying high-risk patients by visualizing signs of hemorrhage in those presenting with acute UGIB. All patients undergoing CE received intravenous metoclopramide at enrollment. Three thoracic sensors were placed in a standard pattern and connected to the DR-2 data recorder, which was attached via a USB cable to a Fujitsu mini-notebook computer running Rapid Viewer. The results showed that seven out of 12 patients (58%) in the CE had high-risk lesions on endoscopy and required emergent therapeutic intervention, while five out of 12 patients in the control group had positive stigmata of hemorrhage at endoscopy [15]. 

Secondary outcomes included the time from enrollment to endoscopy, with patients identified as "high risk" in the CE group averaging 2.5 hours and "low risk" in the CE group averaging 8.4 hours and the control group averaging 8.9 hours. The correlation of CE findings with endoscopic findings showed that all seven "high-risk" patients in the CE group had high-risk lesions on endoscopy. There was no mortality reported in either group. Overall, the CE group demonstrated a shorter time to endoscopy and a higher identification rate of high-risk lesions [15].

In another study, the aim was to determine whether an esophageal capsule could identify a low-risk group of patients with UGIB who could safely wait for elective EGD. The preparation for the CE involved administering 10 mg of metoclopramide intravenously 30 minutes before ingestion, assessing the patient's ability to swallow with 100 mL of water to minimize aspiration risk, and positioning the patient immediately on the right side with a series of gradual elevations of the head of the bed while allowing sips of fluid. The primary endpoints of the study were the detection rate of acute UGIB and the assessment of patients who could have been discharged from the ED based on CE compared to emergency EGD. CE identified a bleeding site in 66% of patients, whereas EGD identified bleeding in 89%, with concordant findings in 55% of cases. CE correctly identified 92% of patients with low-risk lesions suitable for outpatient management, compared to 16% using the Blatchford score. Both triage strategies incorrectly classified one patient with significant endoscopic stigmata as suitable for outpatient EGD. Secondary endpoints included the cost comparison of CE versus EGD, with CE-based triage proving to be cost equivalent overall. For the subgroup with duodenal visualization, CE-based triage resulted in substantial cost savings of $74,404 ($1691 per patient), correctly triaging 92% of patients to outpatient EGD compared to 16% using the Blatchford score [16].

The study by Gralnek et al. aimed to evaluate the feasibility of CE in patients presenting to the ED with acute overt UGIB compared to nasogastric aspiration and EGD. Each patient received a single dose of intravenous erythromycin (250 mg) 30-60 minutes prior to capsule ingestion and wore the standard CE sensor array and data recorder. In terms of identifying gross blood, CE detected blood in the upper GI tract significantly more often (15 out of 18 patients, 83.3%) compared to nasogastric aspiration (six out of 18 patients, 33.3%; p=0.035). CE was particularly effective in detecting fresh blood or coffee grounds in the duodenum, identifying such signs in nine patients where nasogastric tube aspiration was reported as bilious/clear in seven of these nine patients (77.8%) [17].

For detecting peptic/inflammatory mucosal lesions, CE identified 27 out of 40 inflammatory lesions (67.5%), compared with 35 out of 40 (87.5%) detected by EGD (p=0.10, odds ratio (OR): 0.39, 95% confidence interval (CI): 0.11-1.15). When excluding ulcers, CE detected 17 out of 25 lesions (68%) compared with 21 out of 25 (84%) detected by EGD (p=0.39). Regarding the determination of variceal or nonvariceal sources of bleeding, four patients had non-bleeding varices, of which three (75%) were detected by both CE and EGD and one was detected by EGD alone. In evaluating adverse events and technical failures, the video capsule reached the duodenum in 45 out of 46 patients (97.8%). Among the 46 patients, one (2.2%) experienced self-limited shortness of breath, and one (2.2%) had coughing at the time of capsule ingestion. Patient satisfaction was significantly higher with CE compared to EGD and nasogastric tube aspiration [17].

In another study, the aim was to compare the accuracy of the Rockall and Blatchford scores with PillCam ESO® in predicting high-risk endoscopic stigmata. For capsule preparation, all CE patients received intravenous metoclopramide upon enrollment and underwent live-view CE at the bedside with a real-time viewer. Three thoracic sensors were attached in a standard pattern and connected to the DR-2 data recorder, which was linked via USB to a Fujitsu mini-notebook running Rapid Viewer. PillCam ESO 2 was administered following standard procedures [18].

In the group of 12 patients who underwent CE, eight had positive findings confirmed at EGD, while the remaining four had no high-risk stigmata. The mean Rockall and Blatchford scores for patients in the other with high-risk stigmata were 3 (1.5) and 13 (3.1), respectively, compared to 2 (1.6) and 11 (4.2) for those without. In this group, with EGD, the Blatchford score demonstrated 100% sensitivity but 0% specificity, while the Rockall score showed 92.3% sensitivity and 18% specificity. There were no significant differences in the scores between the groups, and no significant associations were observed between the scores and endoscopic findings [18].

Sung et al.'s study aimed to evaluate whether CE could reduce unnecessary hospital admissions in patients with suspected UGIB compared to the standard treatment (ST). Preparation for the CE involved fasting for at least five hours before the procedure and administering a single dose of intravenous metoclopramide (10 mg) 30-60 minutes before capsule ingestion to enhance gastric motility and visualization [19].

The primary outcome measured was the number of patients requiring hospital admission. In the CE group, only seven out of 34 patients (20.6%) were admitted compared to all 34 patients (100%) in the ST group. Secondary outcomes included safety, clinical rebleeding, mortality, and the effectiveness of CE versus the GBS in identifying UGIB patients needing endoscopic intervention. No adverse events were reported related to CE, and only one patient in the CE group experienced a clinical rebleeding event. There was no mortality in either group. CE effectively identified seven patients with significant findings, leading to their admission, whereas the ST group that included the utilization of the GBS showed a higher rate of findings through conventional endoscopy. The study demonstrated that using CE significantly reduced hospital admissions by 76.5% [19].

Marya et al.'s study aimed to compare the effectiveness of early CE versus standard care (SC) in localizing bleeding during hospitalization for new-onset non-hemodynamically significant gastrointestinal bleeding (NHGIB). For CE, patients ingested the capsule within 10 minutes of consent, and real-time monitoring was employed to track the capsule's progression through the GI tract. If the capsule did not pass from the stomach, intravenous metoclopramide or erythromycin was administered, and further monitoring was conducted to confirm bowel transit [20].

The results indicated that the early CE group had a significantly higher rate of bleeding localization, with 27 out of 42 patients (64.3%) achieving this compared to only 14 out of 45 patients (31.1%) in the SC group. Localization of bleeding was predominantly achieved via CE in the early group (92.6%), whereas the SC group primarily relied on EGD (64.3%). Additionally, the early CE group showed a higher rate of diagnosing vascular lesions, with an adjusted odds ratio of 10.73. No significant differences were observed in readmission rates, all-cause mortality, or cumulative hospital costs between the two groups [20].

Hakimian et al. aimed to assess the comparative use of CE as a triaging tool versus SOC for evaluating GI bleeding. For the CE, patients underwent fasting for 6-8 hours before capsule ingestion and swallowed the capsule with 118-237 mL of water without any purgative bowel preparation [21].

The primary endpoint was the detection of active bleeding or stigmata of recent hemorrhage. In the CE group, active bleeding or stigmata of recent hemorrhage were detected in 44 patients (59.5%), compared to 18 patients (25%) in the SOC group, yielding an OR of 5.23 (95% CI: 2.23-12.27). For secondary endpoints, 48.7% of patients in the CE group underwent invasive procedures versus 97.2% in the SOC group (adjusted OR: 0.01; 95% CI: 0.001-0.08). The COVID-19 group also required fewer additional procedures, with a mean of 0.59 (SD 0.77) invasive procedures per patient compared to 1.18 (SD 0.48) in the SOC group (adjusted difference: -0.54; 95% CI: -0.77 to -0.31). There were no statistically significant differences between the groups in rebleeding rates, rehospitalization rates, or transfusion requirements, and no mortality attributed to bleeding was reported in either group [21].

In Meltzer et al.'s study, the aim was to compare the utility of CE versus SC in triaging ED patients with UGIB. Participants randomly assigned to the CE arm received a single dose of intravenous metoclopramide 10 mg at the time of capsule ingestion to promote gastric motility. The primary outcome assessed was the need for hospitalization. The results indicated that hospitalization was significantly lower in the CE group (18.1%) compared to the SC group (76.9%) (p=0.012). In terms of safety measures, no adverse events were reported at day 7 or day 30 follow-up in the CE group, and no missed bleeding lesions were found on follow-up EGD. High patient satisfaction was reported in the CE group. No adverse events related to the need for hospitalization, recurrent bleeding, or complications attributed to the capsule were noted [22].

Akiki et al. conducted a study to evaluate the effectiveness and safety of the PillSense system, a novel swallowed bleeding sensor, for detecting UGIB compared to endoscopic findings. For preparation, patients were required to fast for a minimum of two hours for clear liquids and four hours for other types of food before ingesting the PillSense capsule [23]. 

The study's primary endpoints were the sensitivity and specificity of the PillSense system in detecting blood, as confirmed by subsequent EGD. The PillSense system demonstrated a sensitivity of 92.9% (95% CI: 76.5-99.1%; p=0.02) and a specificity of 90.6% (95% CI: 82.9-95.6%; p<0.001). Secondary endpoints included the positive predictive value (PPV) of 74.3%, negative predictive value (NPV) of 97.8%, positive likelihood ratio of 9.9, and negative likelihood ratio of 0.08. The capsule successfully passed through the GI tract in all patients who underwent the procedure. Safety assessments revealed that 23 out of 126 patients (18.3%) experienced at least one adverse event, but none were related to the PillSense system. The capsule did not obstruct or disrupt the EGD procedure, and there were no serious device-related complications reported [23].

Discussion

The integration of CE into clinical practice, particularly for the evaluation of GIB, represents a significant advancement in diagnostic technology. This review collates data from nine studies evaluating various applications of CE, comparing its efficacy with traditional diagnostic modalities such as endoscopy and nasogastric aspiration. Collectively, these studies highlight the diverse roles of CE, from enhancing triage processes in ED to improving the accuracy of bleeding detection. The findings underscore CE's potential to refine patient management by offering a less invasive and potentially more precise alternative to conventional methods.

In assessing the outcomes of these studies, it becomes evident that CE offers notable advantages in specific scenarios, such as risk stratification and reducing unnecessary hospital admissions. The data reveal a consistent trend where CE either matched or surpassed traditional diagnostic approaches in detecting UGIB, particularly when combined with adjunctive measures like intravenous metoclopramide to enhance gastric motility. This review not only emphasizes the comparative effectiveness of CE in identifying high-risk patients and localizing bleeding but also explores its impact on clinical outcomes, patient safety, and healthcare costs.

General consensus guidelines suggest that an EGD should be performed within 24 hours for patients with suspected UGIB [24]. Nonetheless, limitations in resources and staffing, particularly in rural or community hospitals and during weekends or night shifts, can impede timely access to endoscopic services. Such delays in EGD execution may adversely affect patient outcomes and contribute to prolonged hospital stays and increased healthcare costs [25,26]. An additional advantage of using CE could be the use of less staffing resources. Traditional endoscopic procedures typically require the involvement of multiple personnel, including the endoscopist, technicians, endoscopy nurse, trainees, and anesthesia staff. Additional staff are also needed to assist with the patient's preoperative assessment, equipment processing, recovery, room turnover, and cleaning [27,28].

CE is limited by its passive nature, as it cannot be manipulated once ingested. However, this limitation is minor because the primary objective is not to achieve an exact diagnosis but to stratify risk and triage patients who may require further therapeutic interventions. Additionally, CE is currently a diagnostic tool without therapeutic capabilities, unlike EGD and colonoscopy. Nonetheless, it has been demonstrated that many patients do not require therapeutic interventions and can avoid these invasive procedures, at least in the acute setting; a cohort study showed that 22% of patients seen for UGIB were of low risk and could be treated in outpatiently [19]. 

The GBS is a highly validated and widely used clinical scoring system for triaging patients in need of hospital-based intervention. Recent studies indicate that a GBS of less than 1 or 2 effectively identifies patients with low-risk UGIB, allowing for outpatient management [29,30]. Observational studies have shown that only 11-14% of patients achieve a GBS of 0, which deems them safe for discharge [31,32]. Utilizing GBS as a triage tool can reduce hospital admissions by 15-20% [29]. Conversely, a high GBS at admission is linked to a significant risk of recurrent UGIB post-hospitalization [33]. In this review, we demonstrated that CE can be more effective than GBS in reducing the number of hospital admissions. 

CE presents a promising alternative for the initial assessment and triage of patients with UGIB, especially in settings with limited endoscopic resources. While CE cannot provide therapeutic interventions, its ability to effectively risk-stratify patients allows for timely and appropriate management. Incorporating CE into clinical practice could reduce the burden on endoscopy services and improve patient outcomes by identifying those who truly need urgent intervention versus those who can be managed conservatively. Future research should focus on optimizing the use of CE and exploring its potential integration with other diagnostic tools to enhance patient care further.

In clinical practice, using video CE as a standard method for managing UGIB can improve patient care, especially in places where immediate endoscopy is not available. Training healthcare providers to use CE effectively and creating clear guidelines for its use with other diagnostic and treatment methods are important next steps. Additionally, future studies should compare CE with other triage tools and look at its cost-effectiveness in different healthcare settings. One of the limitations of this review was the heterogeneity in the included studies in terms of designs and the use of various CE types. Another limitation is that it did not utilize a meta-analytic approach in demonstrating the effectiveness of CE.

## Conclusions

The use of CE in the risk stratification and management of UGIB in emergency settings is promising. CE's high diagnostic accuracy can enhance patient outcomes by enabling timely and precise diagnosis, reducing the need for invasive procedures. Future research should focus on larger randomized trials to validate CE's efficacy and explore its cost-effectiveness. Additionally, developing guidelines and training for healthcare providers will be crucial for successfully integrating CE into clinical practice, ultimately improving patient care and optimizing healthcare resources.

## References

[1] Meltzer AC, Bashir S (2015). Promise and potential of video capsule endoscopy in the emergency department. Tech Gastrointest Endosc.

[2] Antunes C, Tian C, Copelin II EL (2024). Upper gastrointestinal bleeding. StatPearls [Internet].

[3] Wuerth BA, Rockey DC (2018). Changing epidemiology of upper gastrointestinal hemorrhage in the last decade: a nationwide analysis. Dig Dis Sci.

[4] Laine L, Barkun AN, Saltzman JR, Martel M, Leontiadis GI (2021). ACG clinical guideline: upper gastrointestinal and ulcer bleeding. Am J Gastroenterol.

[5] Barkun AN, Almadi M, Kuipers EJ (2019). Management of nonvariceal upper gastrointestinal bleeding: guideline recommendations from the International Consensus Group. Ann Intern Med.

[6] Stanley AJ, Laine L (2019). Management of acute upper gastrointestinal bleeding. BMJ.

[7] Lau JY, Yu Y, Tang RS (2020). Timing of endoscopy for acute upper gastrointestinal bleeding. N Engl J Med.

[8] Schembre DB, Ely RE, Connolly JM, Padhya KT, Sharda R, Brandabur JJ (2020). Semiautomated Glasgow-Blatchford bleeding score helps direct bed placement for patients with upper gastrointestinal bleeding. BMJ Open Gastroenterol.

[9] Iddan G, Meron G, Glukhovsky A, Swain P (2000). Wireless capsule endoscopy. Nature.

[10] Liao Z, Gao R, Xu C, Li ZS (2010). Indications and detection, completion, and retention rates of small-bowel capsule endoscopy: a systematic review. Gastrointest Endosc.

[11] Sidhu R, McAlindon ME, Sanders DS, Thomson M (2007). Capsule endoscopy in the evaluation of gastrointestinal disease. Curr Opin Pediatr.

[12] O'Grady J, Murphy CL, Barry L, Shanahan F, Buckley M (2020). Defining gastrointestinal transit time using video capsule endoscopy: a study of healthy subjects. Endosc Int Open.

[13] Ell C, Remke S, May A, Helou L, Henrich R, Mayer G (2002). The first prospective controlled trial comparing wireless capsule endoscopy with push enteroscopy in chronic gastrointestinal bleeding. Endoscopy.

[14] Page MJ, McKenzie JE, Bossuyt PM (2021). The PRISMA 2020 statement: an updated guideline for reporting systematic reviews. BMJ.

[15] Rubin M, Hussain SA, Shalomov A, Cortes RA, Smith MS, Kim SH (2011). Live view video capsule endoscopy enables risk stratification of patients with acute upper GI bleeding in the emergency room: a pilot study. Dig Dis Sci.

[16] Chandran S, Testro A, Urquhart P (2013). Risk stratification of upper GI bleeding with an esophageal capsule. Gastrointest Endosc.

[17] Gralnek IM, Ching JY, Maza I (2013). Capsule endoscopy in acute upper gastrointestinal hemorrhage: a prospective cohort study. Endoscopy.

[18] Gutkin E, Shalomov A, Hussain SA (2013). Pillcam ESO® is more accurate than clinical scoring systems in risk stratifying emergency room patients with acute upper gastrointestinal bleeding. Therap Adv Gastroenterol.

[19] Sung JJ, Tang RS, Ching JY, Rainer TH, Lau JY (2016). Use of capsule endoscopy in the emergency department as a triage of patients with GI bleeding. Gastrointest Endosc.

[20] Marya NB, Jawaid S, Foley A (2019). A randomized controlled trial comparing efficacy of early video capsule endoscopy with standard of care in the approach to nonhematemesis GI bleeding (with videos). Gastrointest Endosc.

[21] Hakimian S, Raines D, Reed G (2021). Assessment of video capsule endoscopy in the management of acute gastrointestinal bleeding during the COVID-19 pandemic. JAMA Netw Open.

[22] Meltzer AC, Limkakeng AT Jr, Gentile NT (2021). Risk stratification with video capsule endoscopy leads to fewer hospital admissions in emergency department patients with low-risk to moderate-risk upper gastrointestinal bleed: a multicenter clinical trial. J Am Coll Emerg Physicians Open.

[23] Akiki K, Mahmoud T, Alqaisieh MH (2024). A novel blood-sensing capsule for rapid detection of upper GI bleeding: a prospective clinical trial. Gastrointest Endosc.

[24] Barkun AN, Bardou M, Kuipers EJ, Sung J, Hunt RH, Martel M, Sinclair P (2010). International consensus recommendations on the management of patients with nonvariceal upper gastrointestinal bleeding. Ann Intern Med.

[25] Guo CL, Wong SH, Lau LH (2022). Timing of endoscopy for acute upper gastrointestinal bleeding: a territory-wide cohort study. Gut.

[26] Lee JG, Turnipseed S, Romano PS (1999). Endoscopy-based triage significantly reduces hospitalization rates and costs of treating upper GI bleeding: a randomized controlled trial. Gastrointest Endosc.

[27] Bai Y, Yao L, Wei T, Tian F, Jin DY, Chen L, Wang M (2020). Presumed asymptomatic carrier transmission of COVID-19. JAMA.

[28] Setti L, Passarini F, De Gennaro G (2020). Airborne transmission route of COVID-19: why 2 meters/6 feet of inter-personal distance could not be enough. Int J Environ Res Public Health.

[29] Stanley AJ, Ashley D, Dalton HR (2009). Outpatient management of patients with low-risk upper-gastrointestinal haemorrhage: multicentre validation and prospective evaluation. Lancet.

[30] Girardin M, Bertolini D, Ditisheim S (2014). Use of Glasgow-Blatchford bleeding score reduces hospital stay duration and costs for patients with low-risk upper GI bleeding. Endosc Int Open.

[31] Laursen SB, Dalton HR, Murray IA (2015). Performance of new thresholds of the Glasgow Blatchford score in managing patients with upper gastrointestinal bleeding. Clin Gastroenterol Hepatol.

[32] Recio-Ramírez JM, Sánchez-Sánchez Mdel P, Peña-Ojeda JA, Fernández-Romero E, Aguilera-Peña M, del-Campo-Molina E, Zambrana-García JL (2015). The predictive capacity of the Glasgow-Blatchford score for the risk stratification of upper gastrointestinal bleeding in an emergency department. Rev Esp Enferm Dig.

[33] Sengupta N, Tapper EB, Patwardhan VR, Ketwaroo GA, Thaker AM, Leffler DA, Feuerstein JD (2016). High Glasgow Blatchford score at admission is associated with recurrent bleeding after discharge for patients hospitalized with upper gastrointestinal bleeding. Endoscopy.

