# Correction: Capsule Endoscopy for the Risk Stratification and Management of Acute Upper Gastrointestinal Bleeding in Emergency Departments: A Systematic Review on Triage, Risk Stratification, and Management

**DOI:** 10.7759/cureus.c370

**Published:** 2025-10-30

**Authors:** Sulaiman M Alamro, Mazi M Alanazi, Wejdan K Suwayyid

**Affiliations:** 1 Department of Medicine, College of Medicine, Qassim University, Buraidah, SAU; 2 Emergency Medicine, King Saud Medical City, Riyadh, SAU

The affiliation for Sulaiman Alamro has been corrected from ‘Emergency Medicine, Qassim University’ to ‘Department of Medicine, College of Medicine, Qassim University’.

